# Supplementary material for: Associations of hypertension and antenatal care-seeking with perinatal mortality: A nested case-control study in rural Bangladesh
Source: PLoS One. 2024 Jul 22;19(7):e0287622. doi: 10.1371/journal.pone.0287622 (PMC11262631; doi:10.1371/journal.pone.0287622)
Supplement: Attachment — Submitted filename: Response to Reviewers Oct 2023.docx [file pone.0287622.s001.docx]

Response to Review

October 28, 2023

Associations of hypertension and antenatal care-seeking with perinatal mortality: A nested case-control study in rural Bangladesh

Allyson P. Bear^1,#a,^*, Wendy L. Bennett^2^, Joanne Katz^1^, Kyu Han Lee^1^, Atique Iqbal Chowdhury^3^, Sanwarul Bari^3^, Shams El Arifeen^3^, Emily S. Gurley^1^

Journal Requirements:

When submitting your revision, we need you to address these additional requirements.

1. Please ensure that your manuscript meets PLOS ONE's style requirements, including those for file naming. The PLOS ONE style templates can be found at

https://journals.plos.org/plosone/s/file?id=wjVg/PLOSOne_formatting_sample_main_body.pdf and 
<https://journals.plos.org/plosone/s/file?id=ba62/PLOSOne_formatting_sample_title_authors_affiliations.pdf>

Response: This has been addressed by the authors.

2. In your Data Availability statement, you have not specified where the minimal data set underlying the results described in your manuscript can be found. PLOS defines a study's minimal data set as the underlying data used to reach the conclusions drawn in the manuscript and any additional data required to replicate the reported study findings in their entirety. All PLOS journals require that the minimal data set be made fully available. For more information about our data policy, please see <http://journals.plos.org/plosone/s/data-availability>.

Upon re-submitting your revised manuscript, please upload your study’s minimal underlying data set as either Supporting Information files or to a stable, public repository and include the relevant URLs, DOIs, or accession numbers within your revised cover letter. For a list of acceptable repositories, please see http://journals.plos.org/plosone/s/data-availability#loc-recommended-repositories. Any potentially identifying patient information must be fully anonymized.

Important: If there are ethical or legal restrictions to sharing your data publicly, please explain these restrictions in detail. Please see our guidelines for more information on what we consider unacceptable restrictions to publicly sharing data: http://journals.plos.org/plosone/s/data-availability#loc-unacceptable-data-access-restrictions. Note that it is not acceptable for the authors to be the sole named individuals responsible for ensuring data access.

We will update your Data Availability statement to reflect the information you provide in your cover letter.

Response: Both the Dryad reviewer link and the citation for publication have been included in the cover letter.

3. Please review your reference list to ensure that it is complete and correct. If you have cited papers that have been retracted, please include the rationale for doing so in the manuscript text, or remove these references and replace them with relevant current references. Any changes to the reference list should be mentioned in the rebuttal letter that accompanies your revised manuscript. If you need to cite a retracted article, indicate the article’s retracted status in the References list and also include a citation and full reference for the retraction notice.

Response: The Reference list has been rebuilt in line with PLOS One formatting requirements. One sentence of the manuscript has been revised (line 229) to bring it current. This resulted in the drop of the reference associated with the old statement and the addition of a reference associated with the new statement (reference number 36 in both the old and the new reference list). The reason for this was not related to retraction, rather due to the length of time that has passed from original drafting of manuscript and publication.

5. Review Comments to the Author

Please use the space provided to explain your answers to the questions above. You may also include additional comments for the author, including concerns about dual publication, research ethics, or publication ethics. (Please upload your review as an attachment if it exceeds 20,000 characters)

Reviewer #1: General Comment:

This study presents a nested matched case-control analysis, utilizing a cross-sectional survey and a demographic database in Bangladesh, to examine the relationship between maternal hypertension and perinatal mortality in rural Bangladesh. The authors propose that a history of hypertension before pregnancy significantly increases the risk of perinatal mortality, acknowledging, however, the lack of direct measurements or medical records to verify self-reported hypertension diagnoses and antenatal care visits. The paper clearly outlines its research procedures and limitations, thereby providing useful insights for readers. Additionally, including interviews with women from rural Bangladesh adds significant value to the study.

Additional Comments:

1. Methods, Cross-sectional survey (L.50-65)
The authors gathered data through interviews instead of questionnaires, despite translating and validating the questions. Given the reference to qualitative research in this paper, it is presumed that the authors considered using qualitative data collection methods during the research design phase. These might include using open-ended questions in interviews or having researchers make field notes during follow-up visits. Such data could offer valuable insights into the participants' perceptions and behaviors versus the reality in Bangladesh and form the foundation for the authors' argument.

2. Discussion (L.204-227)
The authors address the insufficiency of current maternal care in preventing adverse perinatal outcomes and scrutinize why maternal antenatal care behaviors have not effectively functioned as preventive measures (L.199-203). They also hypothesize about women who experienced perinatal deaths and had pre-existing hypertension before pregnancy (L.204-206). Would the interviewers' experiences not be relevant to these causal explanations and assumptions? The reviewer understands that the paper is presented within the framework of an epidemiological study, that is, a quantitative study. However, integrating qualitative research into a quantitative framework could further enhance this study.

Please note that these comments do not constitute a request for manuscript revisions. Thank you for the opportunity to review this quality research paper.

Response: Thanks to the reviewer for the insightful comments and for your review. Per the reviewer’s statement, no revisions were made.

Reviewer #2: Please clarify this DOI <https://doi.org/10.1101/2023.06.13.23291331>.

Response: This DOI links to a pre-print of this manuscript.

What influence did culture and accessibility have on your study variables (health-seeking and perinatal mortality, respectively)?

Response: Thank you for the question. Culture and access to care influence health care seeking in every context, and both are complex factors to understand. This quantitative study was not designed to measure these factors. Qualitative methods would be more effective to understand the effect of socio-cultural influences and decision-making around accessing care.

What are your recommendations for further studies?

Response: The manuscript contains recommendations for further studies in three areas, cited below.

Line 201: Additional research is needed to understand why this contact with the health care system did not have a protective effect.

Line 206-209: Further qualitative research is needed to understand why women with pregnancies complicated by hypertension, including women with a known history of disease, did not have more frequent contact, as per national guidelines, with the health care system in order to effectively manage their high-risk pregnancy.

Line 225-227: These data provide a baseline for future longitudinal research to both investigate the temporality of hypertension and further elucidate how hypertension affects pregnancy outcomes throughout a woman’s childbearing years in rural, resource-constrained settings.
